# Environmental DNA in the Ecological Risk Assessment of Water Pollution: Methods, Applications, Challenges, and Future Perspectives

**DOI:** 10.3390/toxics14070644

**Published:** 2026-07-22

**Authors:** Xiaotian Zhang, Xiaoran Gong, Shanshan Di, Miaomiao Teng

**Affiliations:** 1 School of Energy and Environmental Engineering, University of Science and Technology Beijing, Beijing 100083, China; xtian.zhang@foxmail.com; 2 State Key Laboratory of Environmental Criteria and Risk Assessment, Chinese Research Academy of Environmental Sciences, Beijing 100012, China; 15139405812@163.com; 3 State Key Laboratory for Quality and Safety of Agro-Products/Key Laboratory of Detection for Pesticide Residues and Control of Zhejiang, Institute of Agro-Product Safety and Nutrition, Zhejiang Academy of Agricultural Sciences, Hangzhou 310021, China; dishanshan1018@126.com

**Keywords:** environmental DNA, water pollution, ecological risk assessment, aquatic ecosystem health, molecular biomonitoring, ecological effects

## Abstract

Water pollution and its ecological consequences have become central concerns in watershed governance and aquatic ecosystem conservation. Conventional ecotoxicological research on water pollution has long relied on physicochemical monitoring, laboratory-based single-species exposure tests, and morphology-based biological surveys. Although these approaches have provided essential support for pollutant identification, toxicity characterization, and environmental standard setting, they remain insufficient for resolving community-level responses, food-web perturbations, and ecosystem degradation under multiple-stressor conditions. Environmental DNA (eDNA) has emerged as a promising molecular tool because it is non-invasive, highly sensitive, high-throughput, and capable of detecting multiple taxa simultaneously. In aquatic systems, eDNA applications have expanded from biodiversity detection to pollution diagnosis, ecological health assessment, restoration monitoring, and early warning of ecological risk, while increasingly being integrated with eRNA, multi-omics approaches, machine learning, hydrological modeling, and ecological network analysis. However, several challenges still constrain its broader application, including incomplete methodological standardization, false-positive and false-negative detections, insufficient reference databases, limited quantitative capacity, scale mismatches caused by transport and mixing, and difficulties in causal attribution. This review synthesizes recent progress in the use of eDNA for water-pollution research, with emphasis on its technical workflow, major application domains, integrative analytical frameworks, and methodological boundaries. More specifically, three main points are highlighted: (1) eDNA is shifting water-pollution research from single-species toxicity characterization toward community- and ecosystem-level ecological interpretation; (2) its greatest value lies in its integrative role at the interface of biodiversity monitoring, ecological risk assessment, and management-oriented decision support; and (3) future progress will depend on improvements in standardization, quantitative inference, regional reference databases, and multi-source data integration. Overall, this review clarifies how eDNA can contribute to more robust, ecologically meaningful, and management-relevant assessment of water pollution.

## 1. Introduction

Water bodies are fundamental to ecosystem functioning and human well-being. Under continued industrialization, urbanization, and intensive agricultural development, rivers, lakes, reservoirs, estuaries, and coastal waters are increasingly exposed to persistent and interacting environmental pressures [1,2]. In addition to heavy metals, nutrients, and conventional organic pollutants, emerging contaminants such as pharmaceuticals and personal care products, antibiotics, per- and polyfluoroalkyl substances (PFAS), and microplastics are entering aquatic environments on a continuous basis [3,4,5,6]. Consequently, water pollution is no longer defined simply by elevated concentrations of individual contaminants; rather, it is characterized by source diversity, compositional complexity, and ecologically diffuse risk [7,8].

The ecological consequences of water pollution arise from the interaction of pollutant exposure with habitat alteration, hydrodynamic processes, and biotic interactions [9,10,11]. Accordingly, the focus of water-pollution research has shifted from pollutant identification alone to the interpretation of ecological consequences, including how pollution restructures microbial assemblages, alters trophic relationships, and undermines ecosystem functioning [12,13,14].

Traditional ecotoxicological research on water pollution has mainly relied on three methodological pillars: physicochemical monitoring, laboratory exposure tests, and morphology-based biological monitoring [15]. Physicochemical monitoring can characterize environmental stress, laboratory bioassays can quantify toxic effects under controlled conditions, and morphology-based surveys provide a long-established basis for ecological-status assessment [16]. Nevertheless, these approaches remain limited in complex aquatic environments. Physicochemical measurements do not directly capture biological consequences; laboratory experiments cannot fully reproduce multiple-stressor conditions in the field; and traditional biomonitoring is labor-intensive, highly dependent on taxonomic expertise, and often weak in detecting small-bodied taxa, larval stages, fragments, and cryptic species [17,18].

Against this background, eDNA has emerged as a powerful approach for studying the ecological effects of water pollution [19,20,21]. Available evidence indicates that eDNA metabarcoding can serve as a non-invasive molecular monitoring tool for assessing biodiversity across diverse ecosystems and can substantially broaden the taxonomic scope of community surveys [22,23]. In aquatic systems, eDNA signals are shaped by DNA release, transport, mixing, deposition, and degradation, and therefore represent integrated ecological signals rather than simple snapshots of local communities [24]. These properties—particularly high sensitivity and spatial integration—have enabled eDNA to move beyond species detection and toward ecological risk assessment and biological-effect analysis in polluted aquatic systems.

This review focuses on the role of eDNA in the ecological risk assessment of water pollution, with particular emphasis on its methodological foundations, major application domains, integrative analytical frameworks, and current limitations [25,26]. The central argument advanced here is that the value of eDNA lies not only in improved biodiversity detection, but also in its capacity to connect pollution stress, biological-community change, and ecosystem-level interpretation within a unified analytical framework [27,28]. To clarify the scope and thematic focus of the present review, the literature basis and review structure are outlined in the following section.

## 2. Review Design and Literature Basis

**Figure 1:**
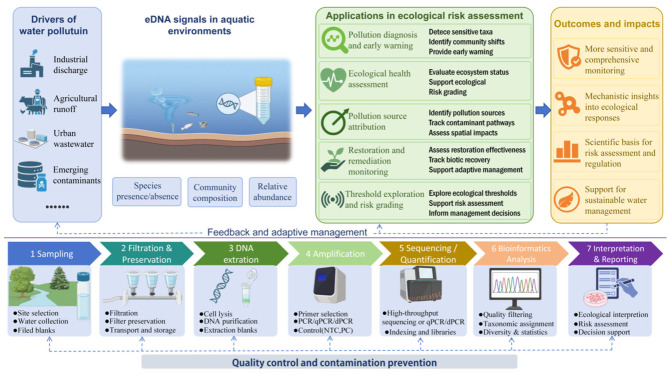
Integrated conceptual and methodological framework of eDNA-based ecological risk assessment for water pollution.

This review was conducted as a critical narrative synthesis of peer-reviewed studies on the application of environmental DNA (eDNA) to water-pollution research, with particular emphasis on ecological risk assessment rather than species inventory alone [29]. To enhance the transparency of the literature basis, a structured search framework was applied using the Web of Science, Scopus, and PubMed databases, supplemented by backward and forward citation tracking of key articles. On the basis of the final bibliography retained in this manuscript, the reviewed literature spans 2015–2026 and is strongly weighted toward recent developments in the field: 87 of the 103 references (84.5%) were published between 2020 and 2026. Search strings combined core concepts related to eDNA and aquatic pollution, including “environmental DNA” OR “eDNA”, “water pollution” OR “aquatic pollution” OR “contaminant”, and “ecological risk assessment” OR “ecological assessment” OR “biomonitoring”, together with thematic extension terms such as “metabarcoding”, “eRNA”, “machine learning”, “hydrological modeling”, “ecological network”, “digital PCR”, and “restoration monitoring”. Studies were retained when they focused on aquatic environments (e.g., rivers, lakes, reservoirs, estuaries, coastal waters, or sediments) and examined eDNA-based approaches in relation to pollution effects, ecological assessment, ecosystem health, restoration trajectories, or risk evaluation [30]. Accordingly, the final reference basis synthesized in this review comprised 103 peer-reviewed publications covering methodological workflow and quality control, pollution diagnosis and ecological assessment, source attribution and restoration monitoring, threshold exploration, and integrative approaches involving eRNA, omics, machine learning, hydrodynamic modeling, and ecological network analysis [31]. Because the present article is intended as a critical narrative review rather than a formal systematic review or meta-analysis, emphasis was placed on assembling a representative, up-to-date, and thematically balanced body of literature directly relevant to ecological interpretation and management-oriented assessment of water pollution. Figure 1 summarizes the overall review framework adopted in this article.

## 3. From Classical Ecotoxicology to Molecular Ecological Monitoring: Why eDNA Matters

Classical ecotoxicology of water pollution is generally structured around the sequence of pollution exposure, biological damage, and risk assessment [32,33]. Its major strengths lie in methodological maturity, analytical clarity, and the relatively good comparability of standardized results. Laboratory toxicity tests provide key parameters such as *LC*_50_, *EC*_50_, and NOEC, which form the basis of ecological risk evaluation and environmental quality criteria [34]. Likewise, benthic invertebrate indices, fish-based integrity indices, and algal or diatom indicator systems have long supported watershed management, environmental impact assessment, and ecological-restoration appraisal [35,36].

However, the methodological boundaries of this framework are increasingly evident. Laboratory toxicity tests are usually performed under controlled conditions using a limited number of model organisms, whereas biological exposure in real aquatic systems is governed simultaneously by hydrodynamics, co-occurring pollutants, nutrient levels, temperature, sediment conditions, and habitat structure [37,38]. In addition, the responses of a single indicator species or a small set of test organisms cannot adequately represent changes occurring at the community or ecosystem level [39,40]. Many forms of ecological degradation are not first manifested as mortality or reproductive inhibition, but rather as dominant-taxon replacement, reduced community evenness, simplification of ecological networks, and erosion of functional redundancy [41,42,43].

Under combined pollution and multiple-stressor conditions, ecological risk assessment is therefore shifting from single-pollutant toxicity characterization toward the analysis of community responses and ecosystem functioning [44,45]. Water pollution is particularly challenging because aquatic systems are both dynamic and connected. In rivers, flow, velocity, tributary inputs, and episodic hydrological events can strongly modify exposure patterns and ecological outcomes [46,47,48]. In lakes and reservoirs, stratification, eutrophication, and sediment resuspension can produce time lags and spatial heterogeneity in biological effects. Pollution responses are further modulated by non-pollutant factors such as dissolved oxygen, substrate conditions, and habitat complexity, so the same pollution level may generate very different ecological consequences under different environmental contexts [49,50].

Within this context, eDNA provides a way to acquire ecological information at greater taxonomic breadth and over broader spatial and temporal scales. A single water sample can capture genetic information from multiple taxa, enabling the construction of higher-density observation networks with minimal disturbance to the environment. This shift reduces dependence on a small number of representative species and makes it possible to interpret pollution-driven changes at the level of whole communities and their interactions. In this sense, eDNA contributes not merely a more sensitive detection tool, but a methodological bridge between exposure evidence, ecological response, and risk interpretation.

However, the transition from conventional monitoring to eDNA does not eliminate bias; it redistributes bias across the analytical chain [51]. Sampling volume and location, particle capture, primer-template mismatch, PCR stochasticity, taxonomic assignment, and hydrological transport can each alter apparent community composition [52]. Technical development should therefore prioritize harmonized sampling, multi-marker validation, replicated controls, transparent bioinformatics, detection-probability modeling, and hydrologically informed interpretation rather than analytical sensitivity alone [53].

To clarify the relative roles of conventional methods and eDNA-based monitoring in ecological risk studies, Table 1 summarizes their principal targets, strengths, limitations, and typical scales of application [54,55].

## 4. Technical Workflow and Key Quality-Control Points for Aquatic eDNA Studies

In aquatic environments, eDNA originates from genetic material released during organismal growth, metabolism, reproduction, excretion, tissue shedding, and post-mortem decomposition [56,57]. It may occur as intracellular DNA, free extracellular DNA, or extracellular DNA adsorbed to suspended particles, biofilms, and sediment surfaces. These different forms vary in stability, transportability, and degradation rate [58,59]. Environmental factors such as temperature, pH, ultraviolet radiation, microbial activity, turbidity, flow velocity, and particulate-matter content can strongly influence eDNA persistence and detectability. Pollutants may further modify these processes by altering water chemistry, microbial metabolism, and particle composition [60,61]. Therefore, eDNA data should be interpreted as integrated ecological signals shaped by both biological activity and environmental processes, rather than as direct proxies for living biomass or local population size.

Accordingly, eDNA concentration or sequence-read abundance should not be interpreted independently of the surrounding water matrix [9]. Temperature and microbial activity regulate degradation [6]. pH, salinity, turbidity, and suspended particles affect preservation and capture [19]. Discharge, stratification, and tidal mixing determine transport and dilution [62]. Under multiple-stressor conditions, the observed signal therefore reflects the combined effects of biological shedding, environmental persistence, particle association, hydrological redistribution, and analytical recovery [63].

Pollutants can modify this chain through both biological and analytical pathways [12]. Heavy metals and other cytotoxic contaminants may alter tissue damage, cell lysis, and organismal activity, thereby changing DNA release [15]. Chronic exposure may also reduce biomass and progressively weaken target signals [17]. Antibiotics may restructure microbial communities and select resistance-associated genetic material, thereby potentially influencing extracellular-DNA turnover [11]. PFAS have been associated with chronic biological stress and community selection, whereas direct experimental evidence for their effects on PCR amplification remains limited [12]. As particulate substrates, microplastics may affect the association and transport of extracellular DNA, although the magnitude of this effect is matrix-dependent [19]. Micro- and nanoplastic exposure may also contribute to changes in aquatic community composition [16]. Co-extracted organic matter, particles, and pollutant-associated compounds could inhibit amplification under some water-matrix conditions [64]. Pollutant-specific calibration and inhibition controls are therefore required before changes in eDNA signal are interpreted as proportional changes in abundance or toxicity [53].

As illustrated in Figure 1, eDNA-based research on the ecological effects of water pollution generally follows a sequential analytical chain, beginning with study design and field sampling and extending through laboratory analysis, bioinformatic processing, and ecological interpretation [62,65]. The reliability of this chain depends not only on laboratory performance, but also on whether sampling design, analytical choices, and interpretative frameworks are matched to the ecological questions being addressed. In this sense, technical workflow and quality control are inseparable from ecological inference [63,66,67].

Sampling design should be tailored to system type and study objective [68,69]. In riverine systems, upstream inputs, tributary confluences, lateral mixing, and temporal variability in discharge should be taken into account, whereas in lakes and reservoirs, depth stratification, seasonal turnover, and sediment resuspension may strongly affect the spatial distribution of eDNA [70,71]. In estuarine and coastal waters, salinity gradients, tidal exchange, and nearshore circulation further complicate signal interpretation. Consequently, site selection, sampling depth, water volume, temporal replication, and field controls must all be defined in relation to hydrological context and expected ecological heterogeneity [72].

eDNA capture is most commonly achieved through filtration or precipitation, with filtration remaining the dominant approach in most aquatic studies because of its compatibility with downstream extraction and amplification [73,74]. However, DNA recovery can vary substantially with membrane material, pore size, filtration volume, water turbidity, and filter clogging [75,76]. Sample preservation is equally important, as DNA degradation may occur rapidly if storage conditions are not properly controlled. Low-temperature storage, freezing, ethanol preservation, and stabilizing buffers are commonly used to reduce degradation and preserve molecular integrity prior to extraction [77,78].

Subsequent laboratory procedures introduce additional sources of variation. DNA extraction should minimize inhibition from humic substances, organic matter, and other co-extracted contaminants [79,80]. Marker selection depends on target taxa and study goals: 12S rRNA and COI are widely used for fishes, COI is frequently applied to benthic invertebrates, 16S rRNA is commonly used for bacterial communities, and 18S rRNA, ITS, or *rbcL* are often used for eukaryotic microorganisms and algae [81,82]. Although single-marker strategies are operationally efficient, they may introduce taxonomic bias and limit ecological coverage. Multi-marker strategies can therefore provide broader taxonomic representation and are often more suitable for studies aiming to characterize pollution-driven changes across multiple trophic levels [83].

After amplification, sequence data require careful quality filtering, denoising, ASV or OTU generation, taxonomic assignment, and contamination screening [64,84]. These bioinformatic procedures are not merely technical steps, but directly influence ecological interpretation. For example, sequence filtering thresholds, reference-database quality, and taxonomic-assignment algorithms can all affect the inferred composition of sensitive and tolerant taxa, thereby shaping conclusions about pollution responses and ecological status [52].

Robust quality assurance is essential if eDNA is to support ecological risk assessment of water pollution [85]. Negative controls are required to detect exogenous contamination, positive controls help evaluate amplification performance, and technical and biological replicates improve confidence in observed patterns [53]. In polluted aquatic systems, additional care is needed to account for transport-driven scale mismatches, temporal lag effects, and misleading signals caused by low DNA concentration or PCR inhibition. Accordingly, quality control should not be viewed as a separate laboratory requirement, but as a core component of study design, data interpretation, and ecological inference.

Overall, the technical workflow of aquatic eDNA studies should be understood as a coupled process linking sampling design, molecular analysis, bioinformatic processing, and ecological interpretation. Only when these components are coherently integrated can eDNA provide robust support for pollution diagnosis, ecological health assessment, restoration monitoring, and risk evaluation in aquatic environments.

## 5. Major Application Scenarios of eDNA in Water Pollution Research

### 5.1. Pollution Diagnosis and Early Warning

Community reorganization is one of the most ecologically meaningful manifestations of pollution stress [86,87]. Compared with single-species toxicity endpoints, community-level responses provide a more integrated view of systemic change under complex pollution scenarios. Because eDNA can simultaneously recover information from multiple trophic levels, it can reveal declines in sensitive taxa, enrichment of tolerant taxa, reduced community evenness, and shifts in overall diversity patterns [88,89].

eDNA is particularly useful for early warning. In urban rivers, industrially affected reaches, mining-impacted waters, and coastal systems exposed to multiple anthropogenic stressors, eDNA can detect community displacement before conspicuous biological collapse becomes evident [51,90]. This capacity is especially valuable in management contexts where subtle ecological deterioration must be identified before severe degradation becomes irreversible.

### 5.2. Aquatic Ecosystem Health Assessment

Assessment of aquatic ecosystem health is a central task in water-environment management. Traditional frameworks typically rely on benthic invertebrates, fishes, algae, and diatoms as indicator groups [91,92]. eDNA can strengthen these frameworks by improving detection efficiency, reducing errors associated with morphological identification, and broadening the range of organisms included in assessment [93,94].

At present, two major pathways can be distinguished. The first is the molecularization of conventional indices, in which eDNA data are used to supplement or partially replace morphology-based observations within established assessment systems [95,96]. The second is the development of genuinely molecular indicators based on OTUs, ASVs, or other sequence-derived features. The former facilitates continuity with existing management practice, whereas the latter takes fuller advantage of the richness of high-throughput molecular data [97]. A combined strategy is therefore likely to provide the most practical route for future monitoring programs.

### 5.3. Pollution Source Attribution and Restoration Monitoring

eDNA further contributes to pollution management by supporting source attribution and restoration evaluation. Effective pollution management requires more than documenting ecological damage; it also requires identifying major pressure sources and determining whether remediation measures have worked [98,99]. Different sources of pollution often leave different ecological fingerprints. Domestic sewage may favor pollution-tolerant taxa, industrial discharges may eliminate sensitive taxa, and agricultural runoff may be associated with nutrient enrichment and shifts in algal or microbial assemblages [100].

Because eDNA supports repeated and minimally invasive sampling, it is especially well suited to restoration monitoring [101]. Rather than focusing only on whether water-quality targets have been achieved, eDNA can reveal whether sensitive taxa are returning, whether biodiversity is recovering, and whether ecological-network complexity is increasing through time [102]. This enables a transition from compliance-oriented assessment to ecologically meaningful evaluation of restoration trajectories.

The same logic is applicable across different restoration settings [101]. In urban rivers, repeated eDNA surveys can test whether pollution-tolerant assemblages are being replaced by more sensitive taxa after sewage interception or habitat improvement [50]. In eutrophic lakes and reservoirs, multi-taxon profiles can track recovery from bloom-dominated states [48]. In estuarine or coastal rehabilitation, eDNA can document recolonization across salinity and pollution gradients [46]. These applications are particularly valuable when pre-restoration baselines and seasonal reference sites are available, because they help distinguish temporary water-quality compliance from sustained biological recovery [98].

### 5.4. Threshold Exploration and Risk Grading

eDNA has also opened new possibilities for threshold exploration and risk grading. Ecological risk assessment ultimately requires thresholds, classes, or warning levels that can support management decisions [103]. Conventional threshold derivation is usually based on single-species toxicity databases, whereas eDNA opens the possibility of identifying turning points in community structure along pollution gradients [14].

At present, however, this application remains exploratory. Community responses are shaped by multiple interacting variables, and sequence read numbers cannot yet be treated as simple quantitative equivalents of abundance, activity, or ecological function. For this reason, eDNA is currently best viewed as a complementary source of evidence that can strengthen threshold exploration when integrated with physicochemical variables, conventional ecotoxicological data, and field ecological observations. The major application domains of eDNA in water-pollution research are summarized in Table 2, together with the types of ecological information they provide and their relevance for environmental management.

A practical risk-grading framework should therefore treat conventional toxicity thresholds and eDNA community breakpoints as complementary rather than interchangeable [12]. The following risk categories are proposed as a conceptual and provisional framework and require further empirical validation before they can be used as regulatory thresholds. LC_50_, EC_50_, and NOEC values provide controlled hazard anchors at the organism level [21]. eDNA-derived change points identify where field communities reorganize along exposure gradients [40]. When both are linked to measured contaminant concentrations and validated with field observations, they can support tiered risk categories [80]. Within this provisional framework, low risk may be indicated where toxicological benchmarks are not exceeded and community displacement is absent [21]. Emerging risk may be indicated by community change before acute thresholds are exceeded [15]. High risk may be indicated by benchmark exceedance together with loss of sensitive taxa or network simplification [47].

## 6. From Biodiversity Detection to Mechanistic Interpretation: Integrative Research Frameworks

To support ecological-effect analysis rather than biodiversity detection alone, eDNA needs to be integrated with other streams of evidence. Physicochemical monitoring identifies pollutant occurrence and concentration, laboratory bioassays provide toxicological responses under controlled conditions, and eDNA reveals how such stressors are reflected in community structure under field conditions [15,17,18]. Considered together, these approaches can establish a more coherent chain linking exposure, ecological response, and risk interpretation [24,40].

A major limitation of eDNA is that it reflects genetic material that is currently present or has recently been present, but not necessarily active biological processes [5,9]. By contrast, eRNA generally decays more rapidly and may therefore provide a closer approximation of current biological activity [5,33,36]. Joint eDNA–eRNA analyses can help distinguish legacy signals from active signals, thereby improving inference about present ecological status [5,33]. For example, a taxon that is strongly represented in eDNA but weakly represented in eRNA may indicate residual DNA persistence with limited contemporary activity, whereas the opposite pattern may suggest an actively responding population [33,36].

Further gains in interpretability can be achieved by integrating eDNA with transcriptomics, metabolomics, metagenomics, and other omics-based approaches [11,12,14]. These methods provide information on gene expression, metabolic disruption, stress pathways, and microbial functional potential, thus shifting interpretation from the question of “who has changed” to the more mechanistically relevant question of “what functional changes are occurring and why” [14,15,40]. This shift is especially important in ecotoxicology, where ecological risk is ultimately defined not only by altered species composition but also by impairment of ecosystem functioning [17,47].

A stepwise toxicological interpretation can be used in complex polluted waters [12]. eDNA first identifies which taxa and community networks have changed [47]. eRNA provides a closer indication of which detected biological components are currently active [5]. Transcriptomic, metagenomic, or metabolomic data can then resolve stress-response genes, resistance functions, and disrupted metabolic pathways [14]. Concordance among these evidence layers strengthens mechanistic attribution [33]. Disagreement among layers can reveal legacy DNA, transient exposure, or delayed community recovery [69].

Machine learning and hydrological modeling offer additional opportunities for strengthening eDNA-based ecological inference. Supervised learning can identify molecular features that best discriminate ecological states or pollution classes, whereas unsupervised learning can reveal latent response patterns and community groupings [61,75,77,80]. Hydrological and hydrodynamic models can, in turn, help correct spatial biases introduced by transport, dilution, and deposition, thereby improving interpretation across heterogeneous aquatic systems [60,62,65,67]. Such integration is particularly important in rivers, reservoirs, and estuarine systems, where transport processes can decouple sampled DNA from local biological communities [49,60,70].

Correction strategies should also differ among water-body types [62]. River studies require upstream-downstream replication, discharge-normalized interpretation, and transport-distance estimates [60]. Lakes and reservoirs require depth-stratified sampling, seasonal-turnover coverage, and explicit treatment of settling and resuspension [65]. Estuarine studies require tidal-phase replication, salinity-stratified sampling, and hydrodynamic correction for bidirectional transport [18]. Applying a single spatial correction model across these systems can create greater bias than it removes [67].

**Figure 2:**
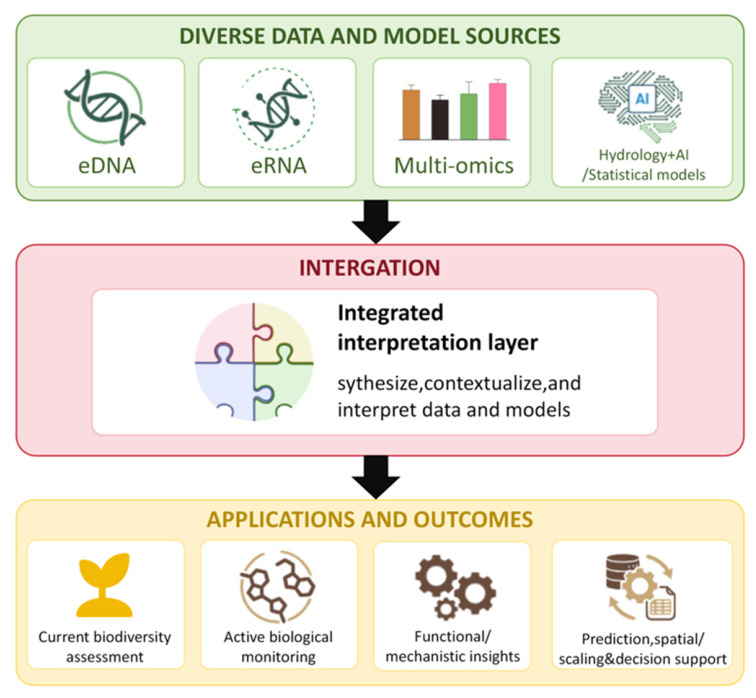
Integrative roadmap from biodiversity detection to mechanistic interpretation and management support.

At a broader systems level, eDNA can also be combined with ecological-network analysis and food-web modeling [35,42]. Although eDNA alone cannot yet reconstruct full trophic networks with high confidence, multi-taxon sequence data can support analyses of network simplification, altered species associations, and reduced system stability under pollution stress [35,47,84]. Collectively, these integrative approaches reposition eDNA from a stand-alone biodiversity tool to a broader analytical platform for ecological risk diagnosis, mechanism-oriented interpretation, and management support [24,73]. Figure 2 summarizes this emerging integrative pathway and highlights the transition from biodiversity detection to mechanistic interpretation and decision-oriented application [77,80].

## 7. Current Bottlenecks and Methodological Boundaries

Despite rapid progress, several methodological bottlenecks continue to constrain the use of eDNA in ecological risk assessment of water pollution. One of the most important is incomplete standardization. Considerable variation persists among studies in sampling volume, filtration method, preservation conditions, primer choice, PCR cycling, and bioinformatic workflow [52,53]. These differences undermine cross-study comparability and complicate interpretation of spatial and temporal gradients [12,52]. In pollution ecology, where biological differences may be subtle, it is often difficult to determine whether observed variation reflects environmental change or methodological inconsistency [51,53].

False-positive and false-negative detections remain additional sources of uncertainty. False positives may result from external contamination, cross-contamination [39,52,53], non-specific amplification, or sequence misannotation, whereas false negatives may arise from insufficient sampling effort, low target-DNA concentration, PCR inhibition, or rapid degradation [59,64,69]. In polluted aquatic systems, such errors can have substantial consequences for ecological interpretation. For example, the detection of a small signal from a sensitive taxon may indicate incipient recovery, but it may also reflect transported DNA from upstream sources; conversely, non-detection may indicate true local loss or simply methodological failure [60,76,82].

Reference-database limitations further weaken taxonomic and ecological interpretation. Although public databases now cover many aquatic taxa, important gaps remain for regionally endemic species, small eukaryotes, and microorganisms [12,19]. Even where barcode sequences are available, ecological traits, pollution tolerance information, and functional attributes are often missing [102]. As a result, sequence identification does not automatically translate into ecological meaning [97].

Quantitative interpretation also remains limited. Sequence read abundance does not scale linearly with biomass, population density, or ecological function, because DNA release rates, environmental decay, and amplification bias differ across taxa and contexts [59,62]. eDNA is therefore currently more robust for identifying relative patterns and community turning points than for direct estimation of abundance or biomass [71]. Although digital PCR, internal standards, and calibration-based approaches are improving quantitative inference, these methods have not yet resolved the broader challenge of translating sequence signals into ecologically meaningful abundance metrics [68,74,79]. As summarized in Table 3, qPCR, ddPCR, internal standards, and hydrological or hydrodynamic models address different components of quantitative and spatial uncertainty and therefore require distinct error-control strategies.

Finally, causal attribution remains particularly difficult under multiple-stressor conditions. Although eDNA is highly effective in describing community change, it cannot by itself determine whether the observed pattern is driven by pollutants, altered flow, habitat degradation, temperature anomalies, or other interacting variables [11,15,40]. In this sense, eDNA is strongest as a source of ecological evidence, but weaker as a stand-alone basis for causal inference [17]. Progress from correlation to causation will therefore depend on tighter integration with controlled experiments, stronger statistical design, and coordinated use of multiple lines of evidence [61,80]. The principal bottlenecks, their implications for interpretation, and possible directions for methodological improvement are summarized in Table 4.

Three complementary designs can improve causal identification [12]. Controlled laboratory or mesocosm experiments isolate pollutant concentration and provide dose-response evidence [16]. Field-gradient surveys test whether the same response occurs under realistic hydrological and habitat variation [40]. Machine-learning or Bayesian models can partition the relative contributions of chemical, hydrological, and habitat predictors [18]. Because variable importance alone does not prove causality, model outputs require independent validation [61]. The strongest inference arises when experimental effects, field gradients, and model-based attribution converge [80].

## 8. Future Research Priorities and Pathways to Management Translation

The future development of eDNA in ecological risk assessment should focus not only on analytical sensitivity, but also on ecological interpretability, regulatory applicability, and practical transferability [52,73]. First, hierarchical standard systems should be established. Research-oriented applications can retain some methodological flexibility, whereas regulatory and long-term monitoring programs require stable procedures and comparable outputs [52,53,85]. A realistic way forward is to develop different levels of standardization for scientific research, routine monitoring, and management implementation, thereby improving comparability without suppressing innovation [53].

International standardization is progressing but remains uneven [52]. European work has focused on validating molecular indices against established ecological-status classes and integrating DNA-based tools with Water Framework Directive-type assessments [100]. United States programs have emphasized assay validation, limits of detection and quantification, contamination control, and transparent reporting [53]. In China, rapid watershed-scale deployment and methodological development have expanded the evidence base [20]. National regulatory protocols, regional reference libraries, and interlaboratory comparability nevertheless remain under development [3]. A proportionate three-level framework is therefore appropriate [52]. Research-grade protocols may retain exploratory flexibility [3]. Routine-monitoring protocols should fix sampling, controls, marker panels, and reporting [52]. Regulatory-grade protocols should additionally require validated decision thresholds, laboratory proficiency testing, chain-of-custody documentation, and auditable uncertainty estimates [53]. Beyond methodological standardization, representative eDNA applications and their management relevance are summarized in Table 5, including basin-scale fish monitoring, urban-river pollution diagnosis, coastal source attribution, restoration assessment, and threshold exploration.

Second, stronger investment is needed in regional reference databases and indicator-taxon libraries. Only databases that reflect local biogeography, dominant pollution pressures, and relevant ecological contexts can support robust and transferable ecological assessment [30,31]. Such databases should not be limited to barcode sequences alone, but should also incorporate ecological traits, functional roles, and pollution-tolerance information [97,102]. This is especially important for understudied aquatic microorganisms and regionally restricted taxa, for which sequence information alone often provides limited ecological meaning [12,14].

Third, future indicator development should move beyond simple species richness or diversity measures. Greater attention should be given to molecular indicators that integrate community composition, key functional taxa, ecological-network properties, and coupling relationships with physicochemical variables [72,73,77]. Such indicators may provide a more mechanistic and ecologically meaningful basis for aquatic ecosystem assessment, especially when linked to validated ecological-quality frameworks [80,94,100].

Fourth, quantitative and model-based approaches require further development. Internal standards, digital PCR, and improved calibration models may strengthen quantitative inference [68,71], while machine learning, Bayesian models, hydrological simulation, and network analysis may improve ecological-state classification, source attribution, and risk prediction [61,75,77,80]. Future work should therefore focus less on isolated methodological refinement and more on integrated frameworks capable of connecting molecular data with ecological interpretation and management decisions [73,77].

Finally, the future value of eDNA will depend on its effective translation into real governance contexts. In watershed management, urban-river restoration, eutrophication control, and coastal ecological rehabilitation, the importance of eDNA lies not in novelty alone, but in its ability to detect ecological change earlier, more accurately, and more comprehensively than many conventional tools [17,25,26,27,28]. Once incorporated into monitoring frameworks, early-warning systems, and restoration-evaluation programs, eDNA has the potential to become a core component of precision water-pollution control and aquatic ecosystem protection [73,98].

## 9. Conclusions and Perspectives

Environmental DNA is reshaping the conceptual and methodological framework of ecological risk assessment for water pollution [24,73]. By enabling broader taxonomic coverage, higher analytical sensitivity, and greater spatiotemporal reach, eDNA is helping water-pollution research move beyond single-species toxicity endpoints toward more integrated analyses of community structure, ecological networks, and ecosystem functioning [35,42]. Based on the literature reviewed here, three major conclusions can be drawn.

First, the scientific value of eDNA lies not only in improved biodiversity detection, but also in its capacity to extend ecological risk assessment from organism-level toxicity evidence to community- and ecosystem-level interpretation [22,24]. In this way, eDNA provides a practical bridge between pollution exposure, biological-community change, and ecological consequence analysis [15,40].

Second, eDNA is most powerful when used as an integrative interface rather than as a stand-alone replacement for conventional monitoring [73]. Its greatest contribution lies in linking physicochemical analysis, biomonitoring, toxicological experimentation, and systems modeling within a more unified framework for ecological risk interpretation [61,80].

Third, the long-term significance of eDNA will depend on its successful transition from methodological promise to management-oriented application [52]. This transition requires stronger standardization, improved regional reference databases, more reliable quantitative approaches, and deeper integration with eRNA, omics-based methods, machine learning, hydrological modeling, and ecological network analysis [5,33,52,53,77,85].

On this basis, four priorities should be emphasized for future research. (1) Standardized protocols should be strengthened across sampling, laboratory procedures, bioinformatic processing, and reporting [52,53,85]. (2) Regional reference databases should be expanded and linked to ecological traits, functional roles, and pollution-tolerance information [97,102]. (3) Greater effort should be devoted to quantitative inference and mechanism-oriented interpretation, particularly through integration with eRNA, multi-omics, and model-based approaches [5,33,68]. (4) eDNA should be translated more effectively into real governance contexts, including watershed management, restoration assessment, eutrophication control, and ecological early-warning systems [25,26,27,28].

With continued progress in these directions, eDNA is likely to evolve from an emerging research tool into a key technical pillar for ecological risk assessment and watershed-scale environmental governance.

## Figures and Tables

**Table 1:** Comparison of conventional methods and eDNA-based monitoring in the ecological risk assessment of water pollution.

Dimension	Physicochemical Monitoring	Single-Species Laboratory Bioassays	Morphology-Based Biomonitoring	eDNA-Based Monitoring
Primary target	Pollutants and environmental variables	Toxic responses of model organisms	Indicator taxa and community structure	Multi-taxon genetic signals in environmental samples
Strengths	Direct measurement of exposure conditions	Standardized toxicity endpoints and strong controllability	High ecological relevance and long management history	Non-invasive, highly sensitive, high-throughput, and capable of simultaneous multi-taxon detection
Limitations	Does not directly reflect biological consequences	Poor representation of complex field conditions and multiple stressors	Time-consuming, labor-intensive, and dependent on taxonomic expertise	Quantitative interpretation remains limited and is affected by database quality and methodological standardization
Typical scale	Site-scale exposure characterization	Individual or population-level toxicity testing	Community-level ecological status assessment	Community- to ecosystem-level monitoring and early warning
Role in this review	Exposure evidence	Toxic-effect evidence	Conventional ecological evidence	Integrated molecular ecological evidence

**Table 2:** Major application domains of eDNA in the ecological risk assessment of water pollution.

Application Domain	Primary Objective	Typical Ecological Information	Management Relevance
Pollution diagnosis and early warning	Identify community displacement under pollution disturbance	Loss of sensitive taxa, enrichment of tolerant taxa, altered community composition	Early detection of ecological deterioration
Aquatic ecosystem health assessment	Evaluate ecological status and biological integrity	Diversity metrics, community integrity, molecular biotic indices	Support watershed assessment and water-quality management
Pollution source attribution	Distinguish ecological response patterns associated with different stress sources	Characteristic taxon combinations and source-specific shifts	Assist source tracking and stress attribution
Restoration monitoring	Assess trajectories of biological recovery during remediation	Return of sensitive taxa, increasing biodiversity, recovery of network complexity	Evaluate whether ecological recovery is occurring rather than simple compliance
Risk early warning and threshold exploration	Identify turning points in community response and changes in risk level	Community replacement and diversity breakpoints along pollution gradients	Provide supporting evidence for ecological thresholds and risk classification

**Table 3:** Comparison of quantitative and transport-correction approaches for eDNA-based ecological assessment.

Method	Best-Suited Use	Main Advantage	Principal Limitation	Error-Control Requirement
qPCR	Target-species or gene quantification across moderate concentration ranges	Mature, relatively low-cost, and high-throughput	Depends on standard curves and is sensitive to inhibition	Standard-curve efficiency, inhibition tests, technical replicates, and LOD/LOQ reporting
ddPCR	Low-abundance targets and absolute copy-number estimation	No external standard curve and comparatively robust at low copy number	Higher cost, lower throughput, and threshold-setting sensitivity	Unified droplet thresholds, positive/negative controls, replicate wells, and uncertainty reporting
Internal standards	Correction of extraction loss, amplification inhibition, and run-to-run variation	Tracks process efficiency across the analytical chain	May not mimic every natural template and can compete during amplification	Defined spike concentration, recovery-rate reporting, and matrix-specific validation
Hydrological/hydrodynamic models	Transport, dilution, deposition, and source-location correction	Improves spatial interpretation beyond the sampling point	Strong dependence on flow data, model assumptions, and calibration	Concurrent discharge/tide data, particle-behavior parameters, and independent spatial validation

**Table 4:** Major bottlenecks in applying eDNA to the ecological risk assessment of water pollution.

Key Issue	Main Manifestation	Implications for Interpretation	Potential Solutions
Insufficient standardization	Large differences in sampling volume, filtration, primers, and bioinformatic workflows	Poor comparability among studies and monitoring programs	Develop end-to-end standard frameworks and minimum reporting standards
False positives and false negatives	Contamination, non-specific amplification, low target concentration, PCR inhibition, and insufficient sampling	Misidentification of species presence, absence, or recovery	Strengthen controls, replication, contamination prevention, and study design
Incomplete reference databases	Missing barcodes, misannotations, and poor coverage of native or understudied taxa	Limited taxonomic assignment and weak ecological interpretation	Build regional databases and link barcodes to ecological trait information
Limited quantitative capacity	Sequence read counts do not map directly onto true abundance or biomass	Difficult to infer biomass, density, or functional intensity	Develop internal standards, digital PCR, and improved quantitative models
Spatial scale mismatch	Signals influenced by transport, mixing, and deposition	Potential mismatch between sampled DNA and local community status	Combine eDNA with hydrological models and spatially explicit sampling designs
Difficult causal attribution	Community change reflects multiple interacting stressors	Pollution-effect pathways remain uncertain	Integrate physicochemical monitoring, experimental validation, and statistical inference

**Table 5:** Representative eDNA application cases and their management relevance.

Application Scenario	Representative Evidence	Observed eDNA Output	Management Use
Basin-scale fish monitoring	Yangtze River basin fish-community assessment	Spatially resolved fish composition and diversity patterns across a large river network	Identifies biodiversity hotspots and supports basin-scale conservation prioritization
Urban-river pollution diagnosis	Urban-effluent effects on freshwater biodiversity and community networks	Taxonomic displacement and altered association-network structure along effluent influence	Separates chemically compliant sites from locations with persistent biological disturbance
Coastal source attribution	Coastal fish assemblages exposed to salinity variation and agricultural runoff	Community patterns associated with both natural salinity gradients and anthropogenic runoff	Supports multi-pressure source interpretation rather than single-variable attribution
Restoration and ecological-status assessment	River ecological-status assessment using diatom metabarcoding	Recovery of sensitive taxa and molecular biotic-index responses	Evaluates biological recovery and continuity with established assessment classes
Threshold and risk exploration	Multitrophic community and network responses to environmental stressors	Community replacement, interaction-network simplification, and stress-associated breakpoints	Provides supporting evidence for warning levels when combined with chemistry and toxicology

## Data Availability

No new data were created or analyzed in this study. Data sharing is not applicable to this article.

## Abbreviations

The following abbreviations are used in this manuscript:

12S rRNA	12S ribosomal RNA
16S rRNA	16S ribosomal RNA
18S rRNA	18S ribosomal RNA
ASV	Amplicon sequence variant
COI	Cytochrome *c* oxidase subunit I
ddPCR	Droplet digital polymerase chain reaction
DNA	Deoxyribonucleic acid
*EC*_50_	Median effective concentration
eDNA	Environmental DNA
eRNA	Environmental RNA
ITS	Internal transcribed spacer
*LC*_50_	Median lethal concentration
LOD	Limit of detection
LOQ	Limit of quantification
NOEC	No-observed-effect concentration
OTU	Operational taxonomic unit
PCR	Polymerase chain reaction
PFAS	Per- and polyfluoroalkyl substances
qPCR	Quantitative polymerase chain reaction
*rbcL*	Ribulose-1,5-bisphosphate carboxylase/oxygenase large-subunit gene
